# Application of knowledge-driven spatial modelling approaches and uncertainty management to a study of Rift Valley fever in Africa

**DOI:** 10.1186/1476-072X-5-57

**Published:** 2006-12-10

**Authors:** Archie CA Clements, Dirk U Pfeiffer, Vincent Martin

**Affiliations:** 1 Epidemiology Division, Department of Veterinary Clinical Sciences, Royal Veterinary College, Hatfield, Hertfordshire, UK; 2 Division of Epidemiology and Social Medicine, School of Population Health, University of Queensland, Herston, Queensland, Australia; 3 Animal Health Service, Food and Agriculture Organisation, Rome, Italy

## Abstract

**Background:**

There are few studies that have investigated uncertainties surrounding the scientific community's knowledge of the geographical distribution of major animal diseases. This is particularly relevant to Rift Valley fever (RVF), a zoonotic disease causing destructive outbreaks in livestock and man, as the geographical range of the disease is widening to involve previously unaffected regions. In the current study we investigate the application of methods developed in the decision sciences: multiple criteria decision making using weighted linear combination and ordered weighted averages, and Dempster-Shafer theory, implemented within the geographical information system IDRISI, to obtain a greater understanding of uncertainty related to the geographical distribution of RVF. The focus is on presenting alternate methods where extensive field data are not available and traditional, model-based approaches to disease mapping are impossible to conduct.

**Results:**

Using a compensatory multiple criteria decision making model based on weighted linear combination, most of sub-Saharan Africa was suitable for endemic circulation of RVF. In contrast, areas where rivers and lakes traversed semi-arid regions, such as those bordering the Sahara, were highly suitable for RVF epidemics and wet, tropical areas of central Africa had low suitability. Using a moderately non-compensatory model based on ordered weighted averages, the areas considered suitable for endemic and epidemic RVF were more restricted. Varying the relative weights of the different factors in the models did not affect suitability estimates to a large degree, but variations in model structure had a large impact on our suitability estimates. Our Dempster-Shafer analysis supported the belief that a range of semi-arid areas were suitable for RVF epidemics and the plausibility that many other areas of the continent were suitable. Areas where high levels of uncertainty were highlighted included the Ethiopian Highlands, southwest Kenya and parts of West Africa.

**Conclusion:**

We have demonstrated the potential of methods developed in the decision sciences to improve our understanding of uncertainties surrounding the geographical distribution of animal diseases, particularly where information is sparse, and encourage wider application of the decision science methodology in the field of animal health.

## Background

Uncertainty is a major feature of human and animal health decision-making and increasing attention is being paid to methods that detect, measure and reduce uncertainty in a range of settings. Uncertainty can be any error, ambiguity or variation in a decision process or the data on which the decision process is based [1]. Uncertainty is particularly apparent in the relatively data-starved environment of tropical health – nowhere more so than on the African continent. Inadequate demographic data in combination with variable disease surveillance activities create an incomplete knowledge of the distribution, epidemiology and impact of a range of tropical diseases.

Recent advances in geographical information system and remote-sensing (GIS/RS) technologies have been applied in a wide range of studies of the spatial distribution of tropical diseases and the factors that influence disease patterns. To a lesser extent, geographical studies have also had the objective of improving resource allocation to disease control and surveillance activities [2]. However, the paucity of data often renders traditional model-based approaches to disease mapping impossible to conduct, while the need for producing such maps as policy and resource allocation tools remains stronger than ever. In this study we aim to present a pragmatic approach to disease mapping that can be applied relatively rapidly for directing disease control activities, while maintaining honesty about the different levels and sources of uncertainty in the absence of extensive field data. We illustrate our approach using the example of Rift Valley fever (RVF) in Africa.

In the current study we explicitly considered decision rule uncertainty, which refers to uncertainty in the way parameters are specified and combined in the decision process. In our analysis, the decision frames were whether geographical units (pixels) were suitable or not suitable for the occurrence of endemic RVF or RVF epidemics according to specific criteria. Criteria refer to factors that influence the suitability of a given location. Fuzzy logic can be applied to model decision rule uncertainty, where the possibility of a criterion being satisfied is defined on a continuous scale by a membership function, which can take any shape (e.g. rectilinear, sigmoidal, exponential, etc.).

As RVF distributions are multifactorial, a method needed to be adopted within the context of multiple criteria decision making (MCDM) to combine membership functions for different criteria – one such method is weighted linear combination (WLC). With WLC, the criteria are standardised for comparison on a common scale, weights are applied so that more important criteria are able to exert a greater influence on the outcome, and a weighted average across criteria is calculated for each pixel, giving the final suitability estimates.

MCDM models using WLC to construct the decision rule are fully compensatory models – a low score for a given factor may be compensated by a high score for another factor. We also considered the application of ordered weighted averages (OWA) analysis, which allows manipulation of the degree to which a high score for a criterion can compensate for a low score in another. With OWA, criteria are weighted for a given pixel according to the rank of their suitability scores within that pixel. For a less compensatory model, the lower ranked factors are given a higher relative weight [3].

Many of the factors that are known to influence RVF suitability do so indirectly, via their relationships with vector and host populations. In this study we also considered methods for making inference from indirect evidence. The method of choice was Dempster-Shafer theory (DST) [4,5], a generalisation of Bayes theory that is thought to better represent uncertainty in near-ignorance situations [6]. Inference using this approach involved statements about belief in, and plausibility of, a given pixel being suitable for RVF occurrence given the evidence presented by different climatic and hydrological indicators. In the current study we used DST to investigate suitability of pixels for RVF epidemics.

## Results

Suitability maps are presented on a graduated green-yellow-orange-red scale, with delineations between green/yellow, yellow/orange and orange/red occurring at scores of 140, 150 and 200 (range 0 to 255). For descriptive purposes we interpreted model outputs in the green scale to indicate unsuitability, outputs in the yellow scale to indicate low suitability, outputs in the orange scale to indicate moderate suitability and outputs in the red scale to indicate high suitability.

### Endemic suitability

**Figure 1:**
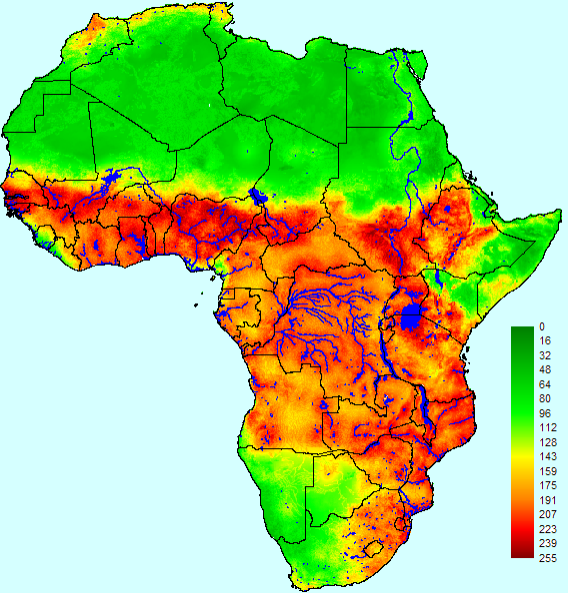
**Endemic suitability map for Rift Valley fever in Africa based on weighted linear combination**. Suitability scores range from 0 (completely unsuitable) to 255 (completely suitable).

With the exception of the coasts of Liberia, Sierra Leone and Cameroon and the arid areas of South Africa, Botswana, Namibia, Kenya, Somalia, Ethiopia, Eritrea and Djibouti, most of Sub-Saharan Africa (SSA) was suitable for the presence of endemic RVF according to our WLC model (Figure 1). The whole of the Sahara was unsuitable, although less arid areas of the Maghreb countries (Morocco, Algeria and Tunisia) were moderately suitable.

**Figure 2:**
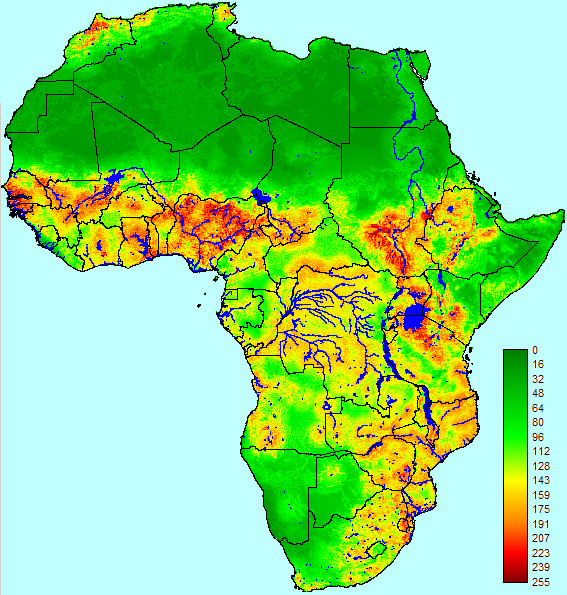
**Endemic suitability map for Rift Valley fever in Africa based on ordered weighted averages analysis**. Suitability scores range from 0 (completely unsuitable) to 255 (completely suitable).

The endemic suitability map derived from our moderately non-compensatory OWA model is presented in Figure 2. The areas that were estimated to be suitable were more limited than in the WLC analysis. For example, both the Central African Republic and Angola were entirely suitable for endemic RVF according to the WLC model, but a large proportion of the area covered by both countries was unsuitable according to the OWA analysis.

### Epidemic suitability

**Figure 3:**
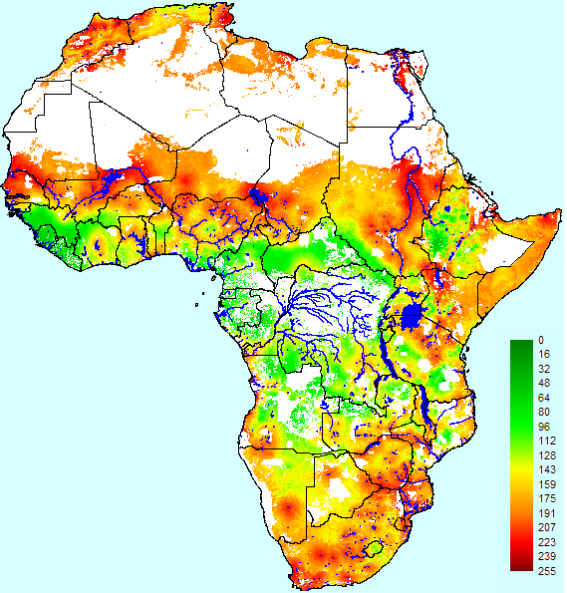
**Epidemic suitability map for Rift Valley fever in Africa based on weighted linear combination**. Suitability scores range from 0 (completely unsuitable) to 255 (completely suitable).

High-suitability areas for RVF epidemics (Figure 3) occurred, according to our WLC model, in the more arid areas of the continent, particularly near the large lakes and rivers, such as the Senegal, Niger and Nile Rivers and Lakes Chad and Turkana, in addition to many other smaller hydrological features. There were a number of high-suitability areas in the Maghreb countries, particularly in Morocco and Tunisia. Low-suitability areas for RVF epidemics included the southern areas of West Africa, all of the high-rainfall tropical areas of Central Africa and the Ethiopian Highlands.

**Figure 4:**
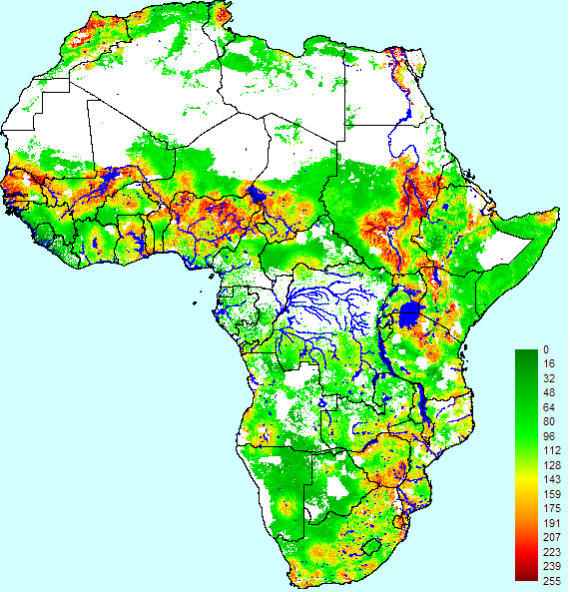
**Epidemic suitability map for Rift Valley fever in Africa based on ordered weighted averages analysis**. Suitability scores range from 0 (completely unsuitable) to 255 (completely suitable).

The epidemic suitability map derived from our OWA model is presented in Figure 4. Moderate and high suitability estimates were more strictly limited than in the WLC analysis to those areas that had a close proximity to hydrological features in the arid areas of the continent.

### Sensitivity analysis

Changing the weights of the lower-weighted factors had little impact on the WLC suitability maps (Table 1), whereas changing the weights of the highest-weighted factors (rainfall in both models) had a bigger, but still not extreme, impact on the suitability estimates. Changing the shape of the relationship between rainfall and endemic suitability from symmetrical to monotonic had the biggest impact. Overall, the models were more sensitive to changes in model structure than to 25% increases or decreases in relative weights between the factors.

**Table 1:** Average changes in endemic and epidemic suitability scores at 10,0000 randomly-selected locations in Africa.

Parameter variation	Average change in suitability score	
	
	Endemic suitability model	Epidemic suitability model
Weight for rainfall increased	10.30	8.84
Weight for rainfall decreased	10.26	8.83
Weight for temperature increased	5.01	0.86
Weight for temperature decreased	5.01	0.89
Weight for elevation increased	1.70	1.58
Weight for elevation decreased	1.70	1.53
Weight for distance to major river increased	2.89	4.19
Weight for distance to major river decreased	2.81	4.10
Weight for distance to minor river increased	1.55	0.96
Weight for distance to minor river decreased	1.49	0.92
Weight for livestock density increased	2.11	1.60
Weight for livestock density decreased	1.99	1.49
Monotonic increasing temperature and rainfall	27.30	-

Parameters of multiple criteria decision making models for Rift Valley fever were varied, including relative weights for factors, which were increased and decreased by 25% of their initial value.

### Validation

**Figure 5:**
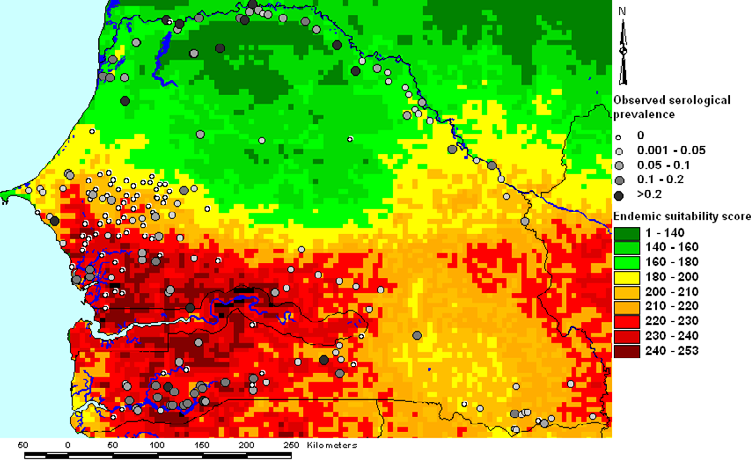
**Overlay of observed serological prevalence and estimated endemic suitability for Rift Valley fever in Senegal**. Suitability estimates were derived using weighted linear combination.

**Figure 6:**
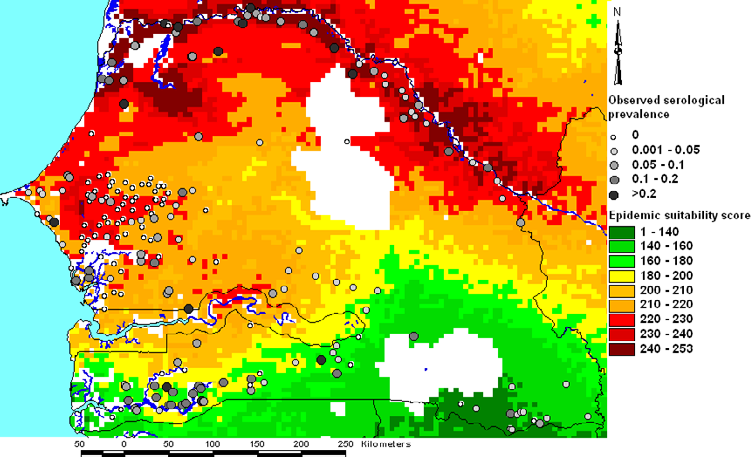
**Overlay of observed serological prevalence and estimated epidemic suitability for Rift Valley fever in Senegal**. Suitability estimates were derived using weighted linear combination.

Overlays of serological prevalence for RVF in ruminant livestock in Senegal and estimated suitability for endemic and epidemic RVF based on our WLC models are presented in Figures 5 and 6 respectively. High serological prevalence was observed in the northern part of the country (the Senegal River basin) and the southern part of the country, particularly in the Cassamance region. This latter area corresponded to an area of high estimated suitability for endemic RVF, whereas the high serological prevalence in the Senegal River basin corresponded to an area of high estimated suitability for RVF epidemics. The intermediate area in central Senegal with low observed serological prevalence was characterised by moderately low suitability for either endemic or epidemic status.

**Figure 7:**
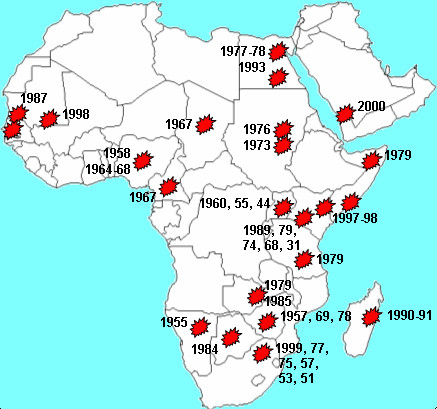
Locations of Rift Valley fever epidemics since the beginning of the 20^th ^century.

Comparison of the epidemic suitability map with the map of known RVF epidemics in Africa (Figure 7) showed that many of the areas with high estimated suitability had experienced epidemics of the disease, such as the Senegal River basin, areas adjacent the Nile in Egypt and Sudan and parts of southern Africa, including South Africa, Namibia and Zimbabwe. A number of areas in SSA had high estimated suitability but had not experienced major epidemics, such as areas adjacent the Niger River and Lake Chad. Additionally, parts of the Maghreb had high estimated suitability but had not experienced epidemics. One important example of an area that did experience a major epidemic that we did not estimate to be highly suitable was north-eastern Kenya. However, we did estimate the neighbouring area around Lake Turkana to be highly suitable for RVF epidemics.

### Dempster-Shafer analysis

**Figure 8:**
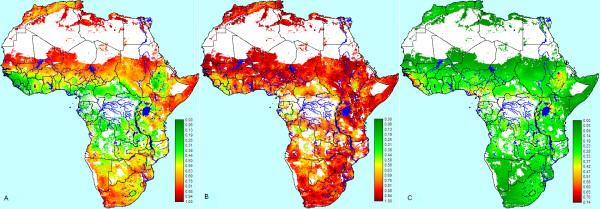
**Outputs of decision-rule uncertainty analysis using Dempster-Shafer theory**. A) Belief in Rift Valley fever (RVF) epidemic suitability, B) plausibility of RVF epidemic suitability and C) belief intervals for RVF epidemic suitability.

The three outputs of the DST model: belief, plausibility and belief interval are presented in Figure 8. Evidence supported the belief that semi-arid areas such as the Sahelian zone of West Africa, the Maghreb countries, Egypt, Sudan, Somalia, northern and north-eastern Kenya, southern Namibia and western South Africa were suitable for the occurrence of RVF epidemics. In addition, the models suggested that it is plausible that many other areas of the African continent were suitable for the occurrence of RVF epidemics, but no strong statement about the belief that these areas are suitable could be made. The areas with the highest belief interval, which were those with the greatest level of uncertainty in terms of whether or not they were suitable for RVF epidemics, included the Ethiopian Highlands, south-western Kenya, southern Nigeria, parts of Cameroon, Sierra Leone and the coastal areas of Guinea and Liberia.

## Discussion

Fuzzy logic, implemented with a WLC framework, has been applied to assist spatial MCDM in a wide range of settings, as divergent as determining the desirability of houses in the real estate industry [7] and prioritising areas for disease vector control [8]. However, there are few examples in the field of human or animal health. Fuzzy logic has been used to define the continental distribution of malaria in Africa, taking into account experimental evidence on the relationships between malaria transmission and temperature and rainfall, and observed malaria transmission patterns in specific climatic zones [9]. A single suitability estimate was calculated, where high suitability was associated with stable endemic transmission and low suitability was associated with malaria epidemics. This contrasts to the current study where we attempted to separate endemic and epidemic occurrence of RVF. However, similarities were evident in that epidemic and endemic areas in the current study were largely exclusive. Also, validation of the malaria suitability map was attempted by visual comparison to existing data sources as in the current study. An expert system based on fuzzy logic was used to model the distribution of a tick species, where the membership functions were derived from a data-driven process of relating tick capture frequencies to a range of climate variables [10]. OWA has also been applied in a range of settings, with one example being an assessment of earthquake vulnerability [11].

A number of common pitfalls in the application of WLC were presented and discussed by Malczewski [12], who stated that the criteria should be measurable and complete (i.e. they cover all relevant aspects of the decision problem). Factor selection on the basis of data availability was criticised. However, comprehensive data on a number of important disease factors are not available for many developing countries (e.g. animal movement data in Africa) and it remains a necessity to select attributes from limited available data resources. We believe that, in the current analysis, most of the important large-scale spatial determinants of RVF ecology were accommodated but as more information becomes available the maps will be updated as part of an ongoing process.

Correlation between attributes was also highlighted as an important issue – Malczewski [12] stated that this results in redundancy due to "double-counting". In our models, temperature and elevation were moderately correlated, however both were kept in the models as the influence of elevation was thought not to be solely related to its association with temperature, but also to its influence on hydrological systems (e.g. low elevations were thought to be more prone to accumulation of ground surface water and flooding, leading to greater risk of RVF epidemics). Slightly lower weights were given to both elevation and temperature to reduce the impact of "double-counting". The issue of spatial scale and levels of aggregation (the modifiable area unit problem) have also been highlighted [12]. In our analysis, we used data that had a common spatial resolution, which was relatively high, and we believe that bias arising from the modifiable area unit problem was likely to be minimal. A possible exception to this is the rainfall variable, which was interpolated using weather station data sampled at different densities across the continent. However, without having the original rainfall data it is impossible to judge what the effects of the modifiable area unit problem was likely to have been for this variable.

We acknowledge that our approach to validation of the maps was qualitative in nature and further work is required for quantitative validation across the African continent. Another major issue with the methods applied in this study is subjectivity, particularly with regards to defining the weights in the WLC models, and the basic probability assignments in the DST analysis. A number of sources of information may be utilised for these purposes, including expert opinion [8], statistical data and published literature [13]. In our WLC analysis we derived crude weights according to the relative frequency by which each factor was mentioned in the published literature. Sensitivity analysis revealed that increasing and decreasing the weights by 25% had a modest effect on the outcome suitability estimates and it appears that correct definition of the model structure and, perhaps, the order of importance of the factors in relation to the objective is more important than achieving a precise estimate of the weights.

There are an increasing number of reports of DST applications in engineering and climatology, such as analyses of water quality [14] and climate change uncertainties [6]. However, there is a dearth of applications in epidemiology, particularly in a spatial context. We have presented, to our knowledge, the first published application of this methodology to a spatial analysis of a disease of veterinary or public health importance.

In the current study, we investigated the suitability of the environment, defined by a number of key parameters, for the occurrence of endemic and epidemic RVF. Environmental suitability for the disease is only one possible consideration when planning allocation of resources to disease surveillance and control activities. Other factors such as the proximity of testing laboratories or other important infrastructure, the distribution of the population at risk, the quality of roads and topography, amongst a wide range of other possible factors, may also be important in determining the cost-effectiveness of disease interventions and surveillance systems. An important area of further research is investigating the applicability of decision sciences to spatially-explicit cost-effectiveness analyses of different resource allocation strategies.

Finally, the question arises as to which of the approaches is best for mapping RVF: WLC, OWA or DST. Each of the approaches is based on different assumptions regarding the epidemiology of RVF. Eastman [1] states that DST or Bayesian modelling are more appropriate than fuzzy logic where the evidence presented in the model criteria is indirect. However, DST is more difficult to implement computationally and, while inference is enriched by presentation of belief, plausibility and belief intervals, this approach has not been widely adopted or tested in the field of spatial epidemiology. A final decision as to which of the approaches is best will depend on wider application of the methodology and more epidemiological data being made available to test the underlying assumptions of each model.

## Conclusion

The current study has demonstrated the potential of methods from the decision sciences, such as MCDM using WLC and OWA, and DST, for enhancing the use of available information in health-related decision making and resource allocation. We believe this is one of the key benefits of these methods: that existing published knowledge as well as expert opinion, accrued over a long period of time and by many individual workers, may be integrated to address the issues of geographical uncertainty with respect to different health problems in sub-optimal environments where field data are not available or too expensive to collect. The outputs from such analyses may be particularly useful for development of control strategies, as they can become available relatively quickly in emergency situations and express suitability as well as its uncertainty. We do, however, urge caution in the interpretation of such maps as estimates may be affected by the necessarily subjective nature of the approach, publication bias and the personal perspectives of the experts from which knowledge was obtained and the analyst who integrates the information to produce the maps, and validation may be difficult or impossible in the absence of supporting data.

We stress that we do not see our mapping efforts as definitive for the geographical distribution of RVF in Africa, but as a valuable step towards the integration of epidemiological knowledge in the context of spatially-defining endemic and epidemic areas of the disease. Refinement of the maps will continue to be an ongoing process as new sources of information arise.

## Methods

### Multiple-criteria decision-making

MCDM involves a sequence of analytical steps. This includes: 1) defining the objective(s), 2) defining the factors (continuous) and constraints (Boolean), 3) defining the relationship between each factor and suitability, 4) standardising the factors so they can be compared, 5) defining the relative importance of each factor in relation to suitability and 6) combining each of the factors and constraints to produce a final weighted estimate of suitability for each location in the study area. We identified two additional steps as being important for determining the credibility of the resultant maps: 7) sensitivity analysis and 8) validation.

### Defining the objectives

The overall analytical objective was to use available published information to better describe the spatial distribution and associated uncertainties of Rift Valley fever at the continental scale. Two specific objectives were identified: 1) to produce estimates of suitability for the presence of endemic RVF activity and 2) to produce estimates of suitability for the occurrence of epidemics of RVF, at all locations in mainland continental Africa. Here, we define an epidemic as a large increase in the number of cases of RVF in animals or animals and man (note: we do not distinguish between epizootics and epidemics), over a large but defined geographical area and a short, defined period of time. We do not consider small, localised outbreaks that may occur in endemic areas, but focus on the massive, devastating epidemics that have caused extensive morbidity, mortality and economic losses in past decades. We define endemic occurrence as stable transmission of RVF as viewed from a large geographical scale, which could occur at a low or a moderately high level, and which could possibly be characterised by small, localised epidemics, but not large-scale epidemics as defined above.

### Defining factors and constraints

In order to define the factors and constraints, the relationship between the factors and suitability and the relative importance of each factor, a systematic review of the published scientific literature was conducted. A wide search was conducted of three on-line databases: CAB abstracts [15], PubMed [16] and ISI web of science [17]. A range of Boolean search terms was used to extract relevant publications. One example was as follows: ("Rift Valley Fever" OR RVF) AND (Spatial OR Geographic* OR Outbreak* OR Epidemic* OR Epizootic* OR Endemic OR Enzootic OR Survey*), where the asterisk "*" represents a root term.

In total, 65 publications with information relating to RVF epidemics and endemic activity were obtained. An electronic database was created in Microsoft Access, for recording information obtained from the selected publications. A list of all factors and constraints that were referred to in the publications was created in the database and queries were run to determine the number of publications that referred to each factor/constraint. The factors were presented in the publications either as observations, expressed opinions or statistical associations. In many cases, observations on the epidemiology of RVF were reported in multiple publications written by the same author or groups of authors or they were made in relation to the same epidemic or serological survey. To prevent multiple inclusion of the same information, only a single entry was made in the database for any observation relating to the same study or made by the same group of authors.

In total, observations were recorded from 47 separate studies/author groups, six studies from North Africa, 10 from East Africa, 10 from Southern Africa & Madagascar, two from Central Africa, 14 from West Africa, two from the Arabian Peninsula and three not specific to a region. Of the 47 separate reports, 25 were epidemic investigations, 19 were serological surveys and three were vector studies. The factors were categorised into those that influenced the distribution of the vector, including rainfall, other climatic factors, topographic and land-cover factors and hydrological factors and those that were related to host suitability, including livestock density, human factors and animal movement (Table 2). A list of the publications used is available from the corresponding author.

**Table 2:** Numbers of publications that reported specific factors as influential for occurrence of Rift Valley fever.

Factor	Number of reports	Epidemic or endemic?	Geographic data coverage available?
Climatic factors:			
Higher rain than normal/floods	17	Epidemic	Indirectly
Annual rainfall	10	Both	Yes
Vegetation/NDVI	6	Both	Yes
Length and timing of rain	4	Epidemic	Indirectly
Temperature	3	Both	Yes
Hydrological factors:			
Proximity to lake/dam	6	Both	Yes
Irrigation	6	Both	No
Dambos/accumulated water	5	Epidemic	Indirectly
Proximity to river	3	Both	Yes
Topographic factors:			
Land-cover type	4	Both	Yes
Low-lying elevation	3	Both	Yes
Ecological zone	3	Both	Yes
Host and vector factors:			
Human factors	10	Epidemic	No
Vector abundance	7	Both	No
Importing infected animals	6	Epidemic	No
Moving livestock to endemic focus	4	Epidemic	No
Breed and other livestock factors	4	Both	No
Sheep present	2	Epidemic	Indirectly
Livestock density	1	Both	Yes
Natural hosts/small mammals	1	Both	No

Data were obtained via a systematic review of the literature. The availability of geographical information system-compatible electronic data for each variable was determined by a wide source of potential sources, including the internet.

### Obtaining geographical data

A search was conducted to obtain data, suitable for inclusion in a geographical information system (GIS), for the spatial factors and constraints identified in the literature search. The following data were obtained: satellite-derived mean land surface temperature (LST) for 1982–1998, obtained from the National Oceanographic and Atmospheric Administration's (NOAA) Advanced Very High Radiometer (AVHRR), elevation, obtained from an interpolated digital elevation model from the Global Land Information System (GLIS) of the United States Geological Survey [18], annual rainfall, interpolated by Texas A and M University [19], perennial and non-perennial water-body locations, obtained from FAOGIS at the Food and Agriculture Organisation, Rome, Italy and livestock density, derived by the Environmental Research Group, Oxford, UK.

The data were imported into the GIS software IDRISI 32 (Clark Labs, Worcester, MA). Surfaces for distance to the nearest major river and distance to the nearest minor river were created in IDRISI. All surfaces were raster-based, with pixel dimensions of 0.05 square decimal degrees (0.05 decimal degrees equals approximately 6 km at the equator), covering an area extending from -17.55 to 51.4 degrees of longitude and -34.85 to 37.35 degrees of latitude, corresponding to the geographical limits of the African continent.

### Defining shapes of the membership functions

The "decision wizard" in the "decision support" menu of IDRISI was used to conduct the WLC analysis (See Eastman [1]). The only Boolean constraint imposed on the suitability maps was livestock density of >0 heads/km^2 ^for epidemic suitability as no epidemics were considered possible where livestock were absent. This constraint was not imposed on the endemic suitability map due to the possibility of a sylvatic component in the endemic cycle.

The shapes of the fuzzy set membership functions for the non-Boolean factors are presented in Table 3. For rainfall, we input a symmetrical relationship with endemic suitability but a monotonic decreasing relationship with epidemic suitability because, in terms of endemic suitability, low rainfall was assumed to be non-conducive to maintenance of stable vector populations and extremely high rainfall was assumed to limit the presence of stable habitats for the developmental stages of the vector population (due to wash-out of ponds, etc.), with intermediate rainfall being optimal, whereas for epidemics, the higher variability of rainfall in arid areas was assumed to be conducive to the precipitation of epidemics and the more consistent rainfall in high-rainfall areas was assumed to be non-conducive.

**Table 3:** Relationships between key variables and suitability for endemic and epidemic Rift Valley fever.

	Endemic suitability			Epidemic suitability		
	
Factor	Shape	Maximum suitability	Minimum suitability	Shape	Maximum suitability	Minimum suitability
Rainfall	Symmetrical	750–2000 mm	<50 mm; >3000 mm	Monotonic decreasing	<10 mm	>3000 mm
Land Surface Temperature	Symmetrical	40 – 45°C	<25°C; >50°C	Monotonic increasing	>40°C	<25°C
Elevation	Monotonic decreasing	<10 m	>2500 m	Monotonic decreasing	<10 m	>2500 m
Distance to major rivers	Monotonic decreasing	<0.1 dec. degrees	> 2.0 dec. degrees	Monotonic decreasing	<0.1 dec. degrees	> 2.0 dec. degrees
Distance to minor rivers	Monotonic decreasing	0.0 dec. degrees	> 0.1 dec. degrees	Monotonic decreasing	0.0 dec. degrees	> 0.1 dec. degrees
Livestock density	Monotonic increasing	>1000 head/km^2^	<10 head/km^2^	Monotonic increasing	>1000 head/km^2^	<10 head/km^2^

One decimal degree equals approximately 120 kilometres at the equator.

For temperature, we input a symmetrical relationship with endemic suitability because the vectors were assumed to have an optimal temperature range, with vector survival being limited by low temperatures and vector life-spans shortened by excessively high temperatures [9]. Intermediate temperatures were assumed to be most conducive to stable vector populations and endemic virus circulation. In contrast, we input a monotonic increasing relationship between temperature and epidemic suitability as viral transmission has been shown to occur more quickly and at a higher rate for higher temperatures [20] and it was assumed that shortened vector life-span at high temperatures would be offset by higher reproductive rates (within the temperature ranges that occur in Africa). Long-term vector survival was assumed not to be a major constraint for epidemics because of the short time periods over which epidemics occur (rapid expansion of the vector population over the short term is the typical pattern with RVF epidemics).

For elevation, distance to major rivers and distance to minor rivers, we input monotonically decreasing relationships with endemic and epidemic suitability as higher elevations and greater distances from water sources were assumed to be non-conducive to either suitability type. For livestock density, we input a monotonically increasing relationship with endemic and epidemic suitability as there would be greater opportunity for host/vector interactions and virus transmission with higher livestock densities.

### Defining thresholds of the membership functions

Table 3 also contains information on the thresholds used to define the membership functions, which represent the values between which linear scaling was performed (outside these thresholds, suitability remained constantly high or low).

Our temperature data was LST, which is usually higher than air temperatures measured above the ground-surface level. As published experimental data involved measurements in temperature-controlled environments, the selection of thresholds based on published literature was not straightforward. We elected to increase the published thresholds by approximately 10 degrees and also widened the optimal temperature range for endemic suitability to allow for multiple vector species with varying temperature requirements.

Unfortunately, no information was available to guide selection of thresholds for the other factors, other than vague observations (low-lying as opposed to elevated; near to a river or lake as opposed to far; high rainfall as opposed to low, etc.). For these variables, the thresholds were necessarily defined according to subjective theoretical reasoning (e.g. minimum suitability was set at the distance from a hydrological features beyond which it was assumed vectors associated with that feature would not reasonably be found), or were set to encompass the whole range of values for that variable, but excluding extreme outliers (e.g. for livestock density, where suitability did not increase above 1000 heads/km^2^).

The factors were standardised on a linear scale using the relationships described in Table 3, where each pixel was given a suitability score on a byte scale ranging from 0 (totally unsuitable) and 255 (totally suitable) according to the value of the factor in that pixel.

### Weighted linear combination

Estimates of the order of importance of each factor were determined by the numbers of publications which reported each factor as being a determinant of suitability for endemic or epidemic RVF activity. Therefore, we gave rainfall the highest weight for both objectives as it was the most commonly reported factor. As no reports presented odds ratios or other measures of relative importance, the actual weights were then assigned somewhat subjectively according to the relative frequency of reporting. The relative differences in weights derived using this approach were subsequently reduced to account for suspected over-reporting of factors that were well-known to be important (particularly rainfall) and the original weights of temperature and elevation were decreased to account for collinearity. The weights, which were normalised to have an additive value of one, are presented in Table 4. The suitability maps were then created in IDRISI using WLC.

**Table 4:** Weights applied to key variables regarding suitability for endemic and epidemic Rift Valley fever.

Factor	Weight: Endemic suitability	Weight: Epidemic suitability
Rainfall	0.45	0.55
Land Surface Temperature	0.18	0.06
Elevation	0.08	0.13
Distance to major rivers	0.15	0.17
Distance to minor rivers	0.07	0.04
Livestock density	0.07	0.05

Ordering and allocation of weights was guided by the results of a systematic review of the published literature.

### Sensitivity analysis

Sensitivity analysis involved varying the structure and weighting values of the MCDM model parameters and measuring the average change in suitability scores at 10,000 randomly selected locations on the map. In terms of structure, the relationships between rainfall and temperature and suitability were input as monotonic increasing in the endemic suitability map, in contrast to the symmetrical relationships in the original model. In terms of weights, the weight of each factor was increased and decreased while the relative weights of the other factors were kept constant, giving a total of twelve subsequent weighted estimates for each location. In the absence of any statistical basis for choosing the amount to increase or decrease the weights, we decided to select 25% of the initial value, as it provided for a wide range of uncertainty while ensuring that the underlying model structure was maintained.

### Validation

Validation of the endemic suitability map was undertaken by comparing the suitability estimates to observed serological data in Senegal. The methods of serological data collection are published in detail elsewhere [21]. Validation of the epidemic suitability map was undertaken by visually comparing the suitability estimates to a map of the locations of known RVF epidemics that occurred during the 20^th ^century, in addition to the observed serological data in Senegal.

### Ordered weighted averages

We hypothesised that the biology of the vectors of RVF may not support a fully compensatory model (e.g. low temperatures may not be offset by high rainfall for endemic suitability as the vector survival will be limited at low temperatures regardless of rainfall). However, RVF virus is known to exist in different ecological systems in South Africa where it has different vector species [22]. Therefore, we also hypothesised that the wide range of possible vector species, which may tolerate different environmental conditions, favours a moderately non-compensatory model over a fully non-compensatory model, justifying the application of OWA. We chose the following weights for the lowest to highest ranked factors for both the endemic and epidemic suitability analysis: 0.29, 0.24, 0.19, 0.14, 0.09 and 0.05 to give a moderately non-compensatory model. The OWA models were implemented in IDRISI.

### Dempster-Shafer analysis

Like Bayesian theory, DST represents knowledge and associated uncertainty using probability distributions; in DST definition of the distributions is termed basic probability assignment (BPA). However, DST differs from the Bayesian approach in a number of key aspects: 1) in DST, probabilities may be assigned to intervals of values (subsets) that may be overlapping, whereas Bayesian probability assignments can only be made to mutually exclusive point values or non-overlapping intervals; 2) belief in a hypothesis in DST is not necessarily the compliment of belief in its negation and 3) ignorance is explicitly accommodated in DST.

In this analysis we considered a single hypothesis with a binary outcome: that a given pixel is suitable for RVF epidemics. Therefore, the "frame of discernement" (the DST analogy of the "decision frame" in decision science terminology) contained two elements: suitable (*A*) and not suitable (*B*). In DST, BPA can relate to belief in *A*, which is the actual support for *A *and plausibility of *A*, which represents the degree to which *A *cannot be disbelieved. The difference between the belief estimate and the plausibility estimate is the belief interval, which represents uncertainty in the acceptance or rejection of *A*.

Eastman [1] states that BPAs are fuzzy measures and we used the fuzzy sets defined in the WLC analysis, scaled to have values ranging from zero to one, as the component BPAs for belief and plausibility. In practice, the decision regarding allocation of sources of evidence (the factors identified in our literature search) to belief or plausibility relates to the estimated strength of the evidence in support of the hypothesis. We assumed that low (and therefore unstable) rainfall and low elevation provided hard evidence for RVF epidemic suitability and therefore combined these elements in the belief estimation. In contrast, we assumed that low temperatures, large distances from major and minor rivers and low livestock density provided soft evidence for RVF epidemic suitability and combined these elements in the plausibility estimation.

The fuzzy set membership functions were integrated using Dempsters rule of combination, which states that the probability assigned to *A *is the sum of the products of the probability assignments of all subsets or values in the component BPAs whose intersections correspond to *A*. We implemented our DST analysis using the "belief" module in IDRISI. For a detailed explanation of how DST is implemented in IDRISI, see Eastman [1] and for an easy to read interpretation of DST see Luo and Caselton [6].

## Competing interests

The author(s) declare that they have no competing interests.

## Authors' contributions

ACAC conducted the systematic literature review, collated the data, conducted the analysis and drafted the paper. DUP supervised ACAC and contributed the original concept of conducting spatial MCDM and DST analyses, which was further developed by ACAC. DUP also revised the manuscript and made many valuable suggestions for changes and improvements. VM provided essential guidance on the epidemiology and control of RVF in Africa and revised the manuscript.
